# Supplementary material for: Interleukin-33-Enhanced CXCR4 Signaling Circuit Mediated by Carcinoma-Associated Fibroblasts Promotes Invasiveness of Head and Neck Cancer
Source: Cancers (Basel). 2021 Jul 9;13(14):3442. doi: 10.3390/cancers13143442 (PMC8306357; doi:10.3390/cancers13143442)
Supplement: Supplementary file 1 [file cancers-13-03442-s001.zip › cancers-1272942-supplementary.pdf]

cancers

MDPI

Supplementary Materials: Interleukin-33-Enhanced CXCR4 Signaling Circuit Mediated by Carcinoma-Associated Fibroblasts Promotes Invasiveness of Head and Neck Cancer

Yu-Chun Lin, Wen-Yen Huang, Tsai-Yu Lee, Yi-Ming Chang, Su-Feng Chen, Yaoh-Shiang Lin and Shin Nieh

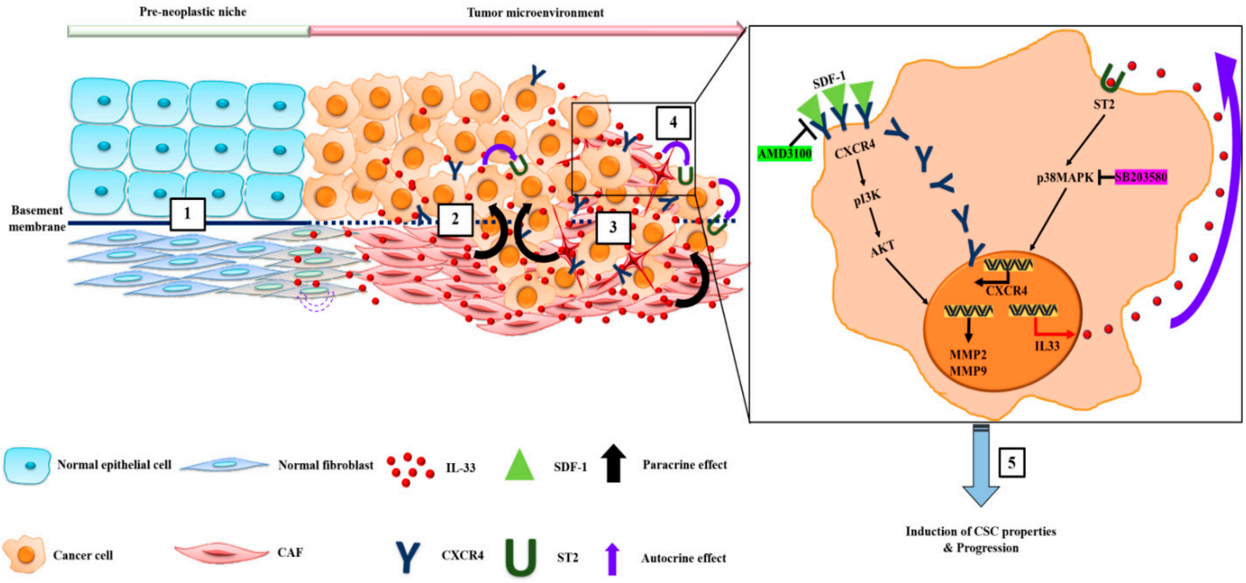

Figure S1. Diagrammatic illustration of five sequential steps explaining how the CAF-induced IL-33-p38 MAPK-CXCR4-SDF-1 loop in the tumor microenvironment contributes to cancer behavior through autocrine and paracrine signaling. The crosstalk between the cancer cells and CAFs is responsible for the progression of the tumor, as shown below. 1. In the preneoplastic state, normal fibroblasts are in an inactive quiescent state and maintain tissue hemostasis. 2. After carcinogenesis occurs, the cancer cells subsequently undergo invasion by breaching the basement membrane, with the generation of CAFs with strong immunoreactivities to CD10 and GPR77 that are transformed from normal precursor fibroblasts at the invasive front. 3. After invasion, cancer cells induce CAFs to produce IL-33, which influences cancer cells through a paracrine effect. 4. Simultaneously, CAF-induced IL-33 reciprocally triggers cancer cells to produce IL-33 in an autocrine fashion and the cancer-secreting IL-33 automatically targets the surface receptor ST2; the subsequent IL-33/ST2 signaling cascade stimulates transcriptional upregulation of CXCR4, thereby creating a newly found "IL-33/CXCR4 regulatory circuit" that undergoes repeated amplification for further activation of SDF-1/CXCR4 signaling transduction. Inhibition of p38 MAPK or CXCR4 using the inhibitors SB203580 and AMD31000 as treatment options may elicit converse results and effectively attenuate tumor progression. 5. Thus, IL-33, through either paracrine or autocrine signaling, plays a functional role in the interactions between CAFs and cancer cells and even in interactions of CAFs and surrounding fibroblasts or other stromal cells. These interactions ultimately lead to advanced progression and induction of CSC properties.

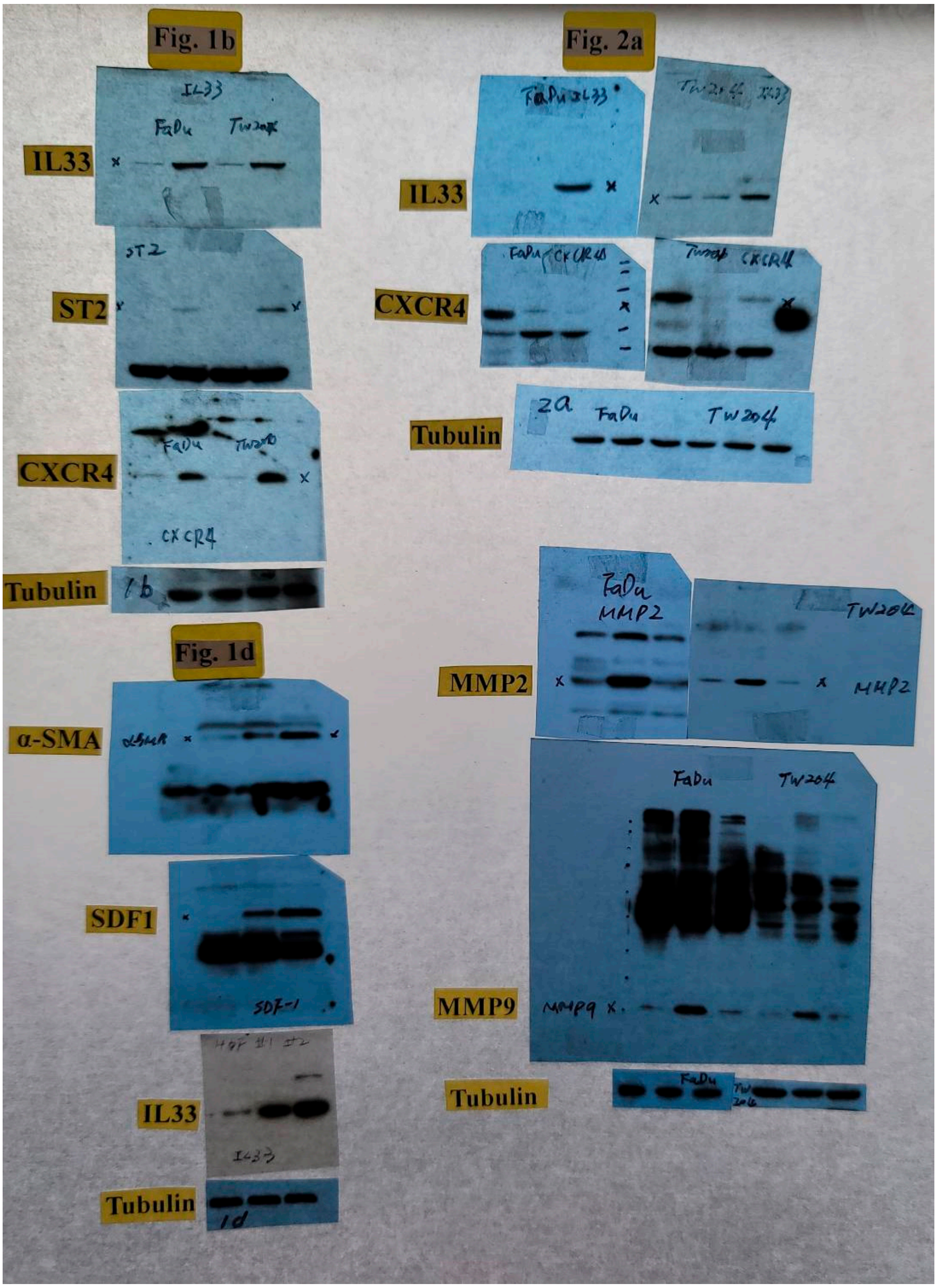
Fig. 1b
IL33
ST2
CXCR4
Tubulin
Fig. 1d
α-SMA
SDF1
IL33
Tubulin
Fig. 2a
IL33
CXCR4
Tubulin
MMP2
MMP9
Tubulin

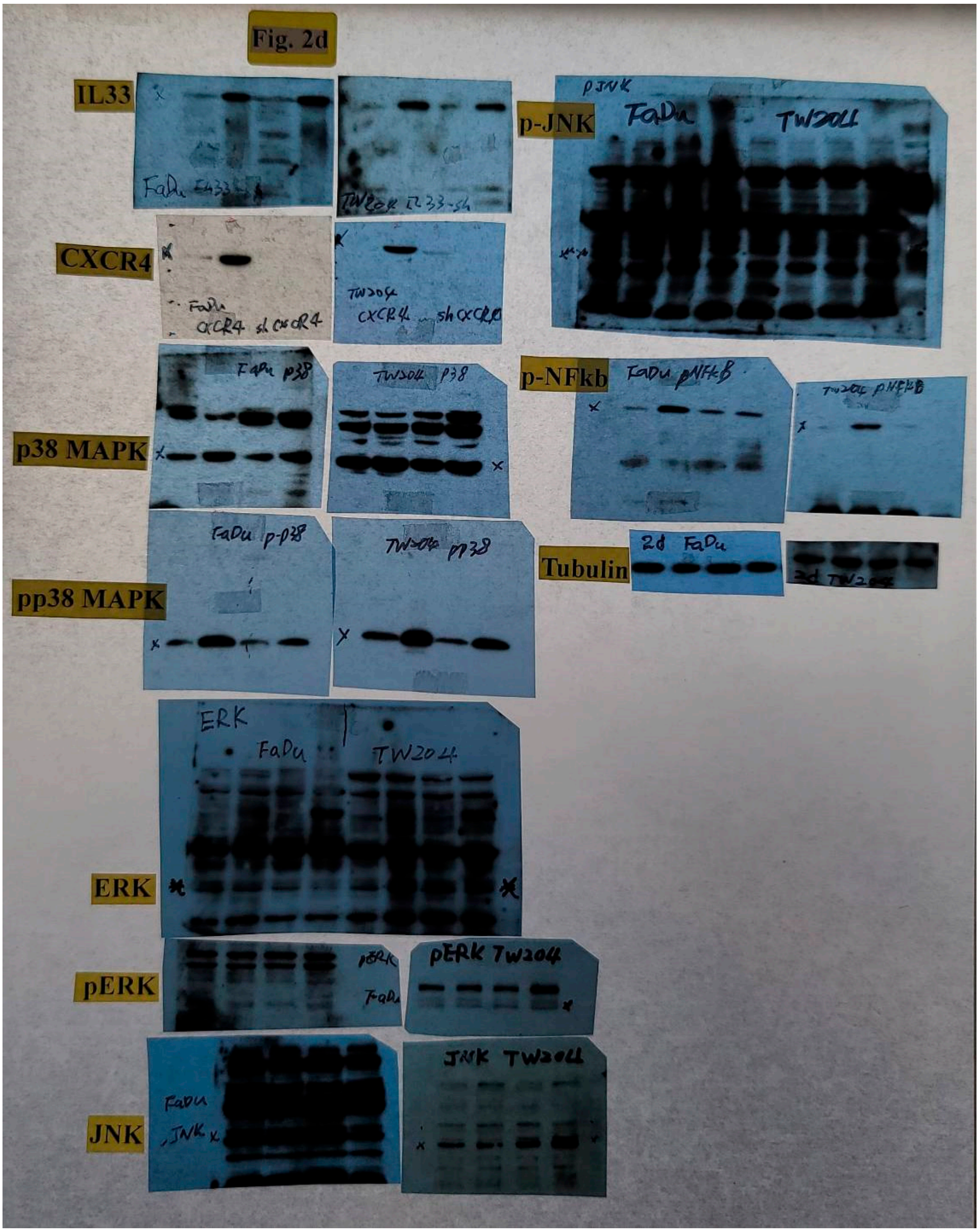
Fig. 2d
IL33
CXCR4
p-JNK
p38 MAPK
p-NFkb
Tubulin
pp38 MAPK
ERK
pERK
JNK

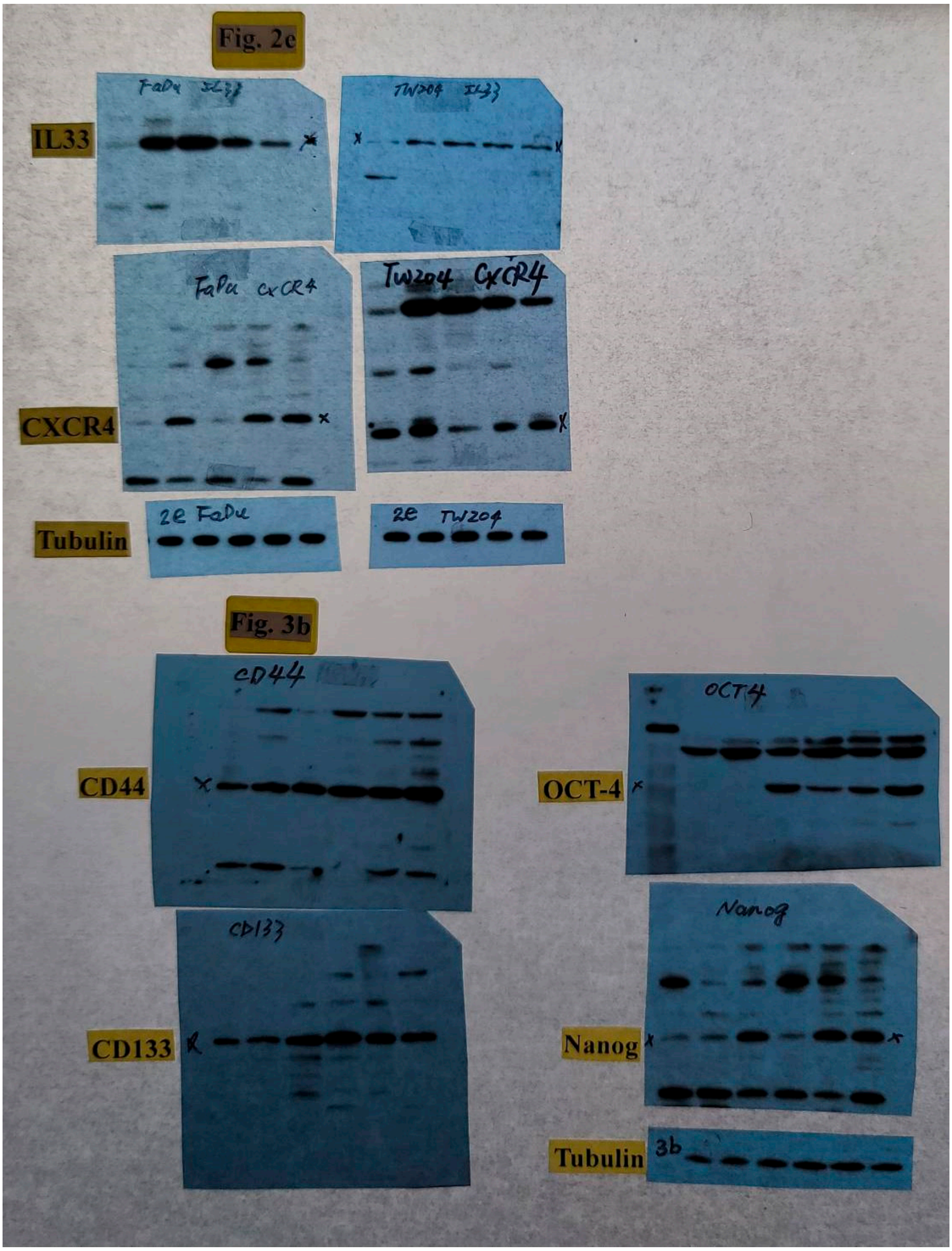
Fig. 2e
IL33
CXCR4
Tubulin
2e FaDu
2e TW204
Fig. 3b
CD44
CD133
OCT-4
Nanog
Tubulin
3b

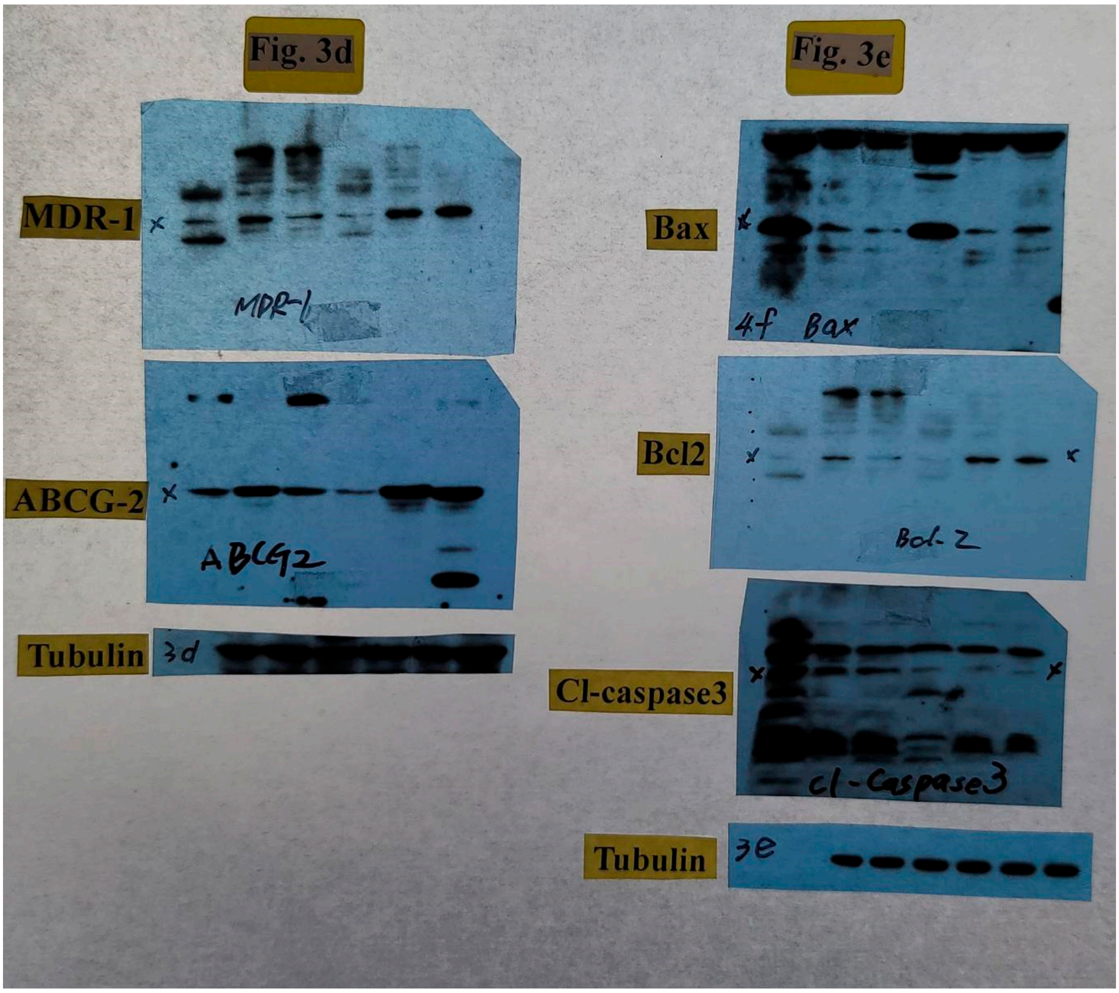

Figure S2. The uncropped Western blots.

Table S1. pMSCV-IL33 coding sequence.

**pMSCV-IL33**
Coding Sequence
> ATGAAGCCTAAAATGAAGTATTCAACCAACAAAATTTCCACAGCAAAGTGGAAGAACACAG-<br>CAAGCAAAGCCTTGTGTTTCAAGCTGGGAAAATCCCAACAGAAGGCCAAAGAAGTTT-<br>GCCCCATGTACTTTATGAAGCTCCGCTCTGGCCTTATGA-<br>TAAAAAAGGAGGCCTGTTACTTTAGGAGAGAAACCACCAAAAGGCCTTCAC-<br>TGAAAACAGGTAGAAAGCACAAAAGACATCTGGTACTCGCTGCCTGTCAACAGCAGTC-<br>TACTGTGGAGTGCTTTGCCTTTGGTATATCAGGGGTCCAGAAATATACTAGAGCAC-<br>TTCATGATTCAAGTATCACAGGAATTTCACCTATTACAGAGTATCTTGCTTCTCTAA-<br>GCACATACAATGATCAATCCATTACTTTTGCTTTGGAGGATGAAAGTTATGAGATATATGTT-<br>GAAGACTTGAAAAAAGATGAAAAGAAAGATAAGGTGTTACTGAGTTACTATGAG-<br>TCTCAACACCCCTCAAATGAATCAGGTGACGGTGTTGATGGTAAGATGTTAATGG-<br>TAACCCTGAGTCCTACAAAAGACTTCTGGTTGCATGCCAACAACAAGGAACACTCTGTG-<br>GAGCTCCATAAGTGTGAAAAACCACTGCCAGACCAGGCCTTCTTTGTCCTTCATAA-<br>TATGCACTCCAACTGTGTTTCATTTGAATGCAAGACTGATCCTGGAGTGTTTATAGGTG-
> Protein Sequence
> MKPKMKYSTNKISTAKWKNTASKALCFKLGKSQQKAKEVCPMYFMKLRSGLMIKKEACYFR-<br>RETTKRPSLKTGRKHKRHLVLAACQQQSTVECFAFGISGVQKYTRALHDSSITGISPIT-<br>EYLASLSTYNDQSITFALEDESYEIYVEDLKKDEKKDKVLLSYYESQHP-<br>SNESGDGVDGKMLMVTL-<br>SPTKDFWLHANNKEHSVELHKCEKPLPDQAFFVLHNMHSNCVSFECKTDPGVFIGVKDNHLA-

Table S2. List of antibodies used in this study.

Protein	Assay	Origin
Interlukin-33	WB/IHC	ALX-804-840, Enzo Life Sciences
CXCR4	WB/IHC	MAB172, R&D system
α-Smooth muscle actin	WB	D4K9N, Cell Signaling Technology
SDF1	WB	ab18919, abcam
ST2	WB/ICC	ab25877, abcam
p38MAPK	WB	GTX27952, Gene Tex
pp38MAPK	WB	D3F9, Cell Signaling Technology
ERK	WB	sc-514302, Santa Cruz Biotechnology
pERK	WB	sc-7383, Santa Cruz Biotechnology
JNK	WB	sc-7345, Santa Cruz Biotechnology
pJNK	WB	sc-6254, Santa Cruz Biotechnology
pNFκB	WB	sc-166748, Santa Cruz Biotechnology
CD44	WB	ab157107, abcam
Cd133	WB	ab19898, abcam
OCT4	WB	D73G4, Cell Signaling Technology
Nanoge	WB	D6D9, Cell Signaling Technology
ABCG2	WB	sc-55510, Santa Cruz Biotechnology
MDR1	WB	D3U1W, Cell Signaling Technology
β-Tubulin	WB	ab15568, abcam

Table S3. List of primers used in this study.

Name	Sequence
IL-6	F: 5′- CAGGAGCCCAGCTATGAACT<br>R: 5′- AGCAGGCAACACCAGGAG
IL-8	F: 5′-CAGAGACAGCAGAGCACACAAGC<br>R: 5′-GAGATGGTTCCTTCCGGTGGT
IL-33	F: 5′-CCACCAAAAGGCCTTCACTGAAA<br>R: 5′-CCAAAGGCAAAGCACTCCACAGT
ST2L	F: 5′- GGGAGAGATATGCTACCTGGAGA<br>R: 5′- CGCCTGCTCTTTCGTATG
CXCR4	F: 5′-GGATATAATGAAGTCACTATGGGAAAA<br>R: 5′-GGGCACAAGAGAATTAATGTAGAAT
MMP2	F: 5′- TTGGCAGTGCAATACCTGAA<br>R: 5′- GAGTCCGTCCTTACCGTCAA
MMP9l	F: 5′- CATCGTCATCCAGTTTGGTG<br>R: 5′- CAGAAGCCCCACTTCTTGTC
GAPDH	F: 5′-TCCACTGGCGTCTTCACC<br>R: 5′-GGCAGAGATGATGACCCTTTT
